# Trends in health behavior and weight outcomes following enhanced afterschool programming participation

**DOI:** 10.1186/s12889-021-10700-4

**Published:** 2021-04-07

**Authors:** Jessica Rieder, Jee-Young Moon, Joanna Joels, Viswanathan Shankar, Paul Meissner, Elicia Johnson-Knox, Bailey Frohlich, Shelby Davies, Judy Wylie-Rosett

**Affiliations:** 1 Division of Adolescent Medicine, Department of Pediatrics, Children’s Hospital at Montefiore, 3415 Bainbridge Avenue, Bronx, NY USA; 2 Department of Epidemiology and Population Health, Albert Einstein College of Medicine, Jack and Pearl Resnick Campus, 1300 Morris Park Avenue, Bronx, NY 10461 USA; 3 Care Management Organization, Montefiore Medical Center, 111 East 210th Street, Bronx, NY 10467 USA; 4 Division of Adolescent Medicine, Children’s Hospital of Philadelphia, 3401 Civic Center Blvd, Philadelphia, PA 19104 USA

**Keywords:** Afterschool programming, School health, Physical activity, Target behaviors, Sleep, Healthy eating, Adolescent obesity, Wellness Cascade

## Abstract

**Background:**

The United States needs to increase access to effective obesity prevention and treatment programming for impoverished youth at risk for health disparities. Although recommended, schools have difficulty consistently implement evidence-based obesity programing. We report on the effectiveness of adding structured nutrition education and minimum physical activity (PA) requirements to standard middle school after-school programming.

**Methods:**

Using a longitudinal pre-post study design, we evaluated program effectiveness at one year on target behaviors on students recruited during three consecutive school years (2016–2018). We used generalized linear (or logistic) mixed-effects modeling to determine: 1) impact on healthy weight and target healthy behavior attainment, and 2) whether target behavior improvement and weight change were associated with after-school program attendance. The seven *target behaviors* relate to eating healthy, physical activity, and sleep.

**Results:**

Over the three years, a total of 76 students enrolled and completed one year of programming (62% Hispanic, 46% girls, 72% with BMI > 85th %ile, 49% with BMI > 95th %ile). Of students with BMI > 85th %ile, 44% maintained or decreased BMI Z-score. There were improvements (non-significant) in BMI Z-score and the adoption of four healthy eating behaviors: fruit, vegetables, sugar-free beverages, and unhealthy snack food. Students with higher after-school attendance (> 75%) had greater improvements (non-significant) in composite behavior scores, BMI Z-score, and in most target behaviors (5/7) than students with lower after-school attendance (< 75%). Sleep improvements were significantly associated with BMI Z-score decrease (Beta = − 0.05, 95% CI (− 0.1,-0.003), *p* = 0.038.)

**Conclusions:**

Enhancement of existing after-school programming with structured nutrition education and minimum physical activity requirements demonstrates positive improvements in several health behaviors and weight outcomes. Adopting enhanced after-school programming increases access to health activities and may bring us closer to solving obesity in at-risk youth in impoverished communities.

**Trial registration:**

ClinicalTrials.gov*identifier (NCT number):* *NCT03565744**. Registered 21 June 2018 – Retrospectively registered.*

**Supplementary Information:**

The online version contains supplementary material available at 10.1186/s12889-021-10700-4.

## Background

The prevalence of obesity in the United States is ~ 1 in 5 in 2–19-year-old children and adolescents [1, 2]. The most severe forms of obesity are increasing, especially among adolescents and non-Hispanic blacks [3]. Children with low socioeconomic status (SES) are 1.4 times more likely to be obese than higher SES children, and relative to higher SES Caucasian adolescents, low SES ethnic minority youth are less likely to live in neighborhoods supportive of physical activity [4, 5]. Adolescents living in low-income urban environments have limited exposure to opportunities that can reduce health disparities compared with other youth [6]. Simulation models indicate that childhood overweight and obesity will continue to be a significant health problem in the United States and that obesity in adolescence increases the risk for many chronic conditions [7]. Despite the risk of health disparities and more severe forms of obesity for minority youth, in particular, there are limited effective preventive and treatment interventions for these youth [8–10].

The U.S. Preventive Services Task Force (USPSTF) recommendations to promote improvements in weight status and cardiovascular and metabolic risk factors include referral of children and adolescents with obesity to comprehensive, intensive behavioral interventions that are moderate to high intensity (> 26 contact hours over 2–12 months) [8]. Depending on the degree of overweight or obesity, staged interventions with increasing intensity of care provided by a multidisciplinary team of medical providers, nutritionists, mental health providers, exercise specialists, and health coaches are recommended and include physical activity, nutrition and sedentary behavior reduction education, parental involvement, and cultural tailoring [9, 11, 12]. While such intensive weight management programs effectively reduce weight and risk for developing type 2 diabetes mellitus (DM) [9, 12–14], they tend to be located in highly specialized treatment centers, delivered by highly trained staff, and require intensive patient engagement [15].

Large-scale schools or community public health efforts targeting youth tend to be less intensive and focus on obesity prevention rather than addressing the needs of youth with obesity and severe obesity. While they may effectively engage and promote healthy lifestyle behaviors for large numbers of youth, they are often not comprehensive in nature, and many have not shown improvements in BMI [9, 16–18]. Further, the Community Preventive Services Task Force (CPSTF) has found insufficient evidence to recommend school-based obesity programs to prevent or reduce overweight and obesity among children and adolescents [19]. In a cross-sectional survey of schools conducted in 2016, Kenney et al. found that slightly less than half (*n* = 117, 47.4%) of schools surveyed offered any obesity prevention program; only 17 (6.9%) reported using a predeveloped program, and 7 (2.8%) reported using a program with evidence for effectiveness [20].

Afterschool settings have focused on increasing physical activity and nutrition education have predominantly concentrated on the general population of middle school children, without highlighting a specific ethnicity, baseline obesity, or socioeconomic background, and have not reported significant improvement in BMI or measured lifestyle changes [21–24]. Findings from other interventions provide guidance for improving physical activity, related to the amount and quality of after-school programming required, and document the degree to which after-school programs increase physical activity or access to fruits and vegetables [25, 26]. Yet, the effectiveness of these interventions has not been reported. The degree to which the findings of these interventions can be applied and translated to predominantly at-risk, low SES minority youth living in low-income urban environments for health disparities youth is not evident.

The increased costs of interventions and health care in resource-limited environments have fueled the demand for applicable evidence for effective weight management programs integrated into existing school and after-school programming environments. Further, there is a need for programs that decrease weight gain trajectory while addressing the needs of high-risk, low SES youth with obesity and severe obesity and at most risk for disparities. We piloted the *B’N Fit POWER* initiative, a middle school-based comprehensive wellness program for low-income minority urban youth in partnership with a school and its onsite stakeholders. This initiative integrates weight management programming into existing onsite school-based health center clinical services and after-school programming [27]. Our initial implementation strategies are reported elsewhere [27]. This paper reports on the effectiveness of adding structured nutrition education and a minimum requirement for physical activity to standard middle school-based after-school programming.

## Methods

### Study design and objectives, and study population

We conducted a longitudinal study to evaluate the B’N Fit POWER initiative’s effectiveness on target behavioral attainment and weight reduction in a real-world setting and maximize its generalizability. Study children for this evaluation were recruited between 2016 and 2018. Most of the students participated and followed for a full academic year. Each student served as his or her own control with target behavior adoption measured four times over the academic year (0, 3, 6, and 9 months). The primary aims were to evaluate the program’s impact on healthy weight and target healthy behavior attainment and whether target behavior improvement and weight change were associated with after-school program attendance measured by BMI-Z-score. The Montefiore–Einstein Institutional Review Board (IRB) approved the study. Parents received an opt-out consent letter for health screenings and provided IRB approved written or verbal consent (if written consent was not feasible to obtain) and HIPAA authorization during program enrollment, and students provided assent at their initial School-Based Health Center *(*SBHC) visit, which is described in detail elsewhere [27].

### Setting

The study is set in the Bronx, NY, the nation’s poorest urban county, consistently New York State’s least healthy county, and a predominantly minority community (56% Hispanic, 44% African-American) [28]. The Bronx has the highest proportion of children living below poverty in the United States with 21% of children below age 6 in deep poverty [29]. Among these populations, school absenteeism is high, school completion rates are low (71%), and unemployment is high (25%). The neighborhood residents have low access to recreation and facilities (3.25 facilities per 100,000 residents), and 77% of Bronx residents are likely to be living in a geographic area designated as a “Health Professional Shortage area.” These issues may likely contribute to low health care access and more health issues [30–32]. At a prevalence rate of 22.3%, rates of obesity among over 240,0000 school-age children in the Bronx are higher than other boroughs in NYC and higher than the national estimates [28, 33–35].

### B’N fit POWER initiative

Set in *Public School/Middle School-95 (PS/MS 95), B’N Fit POWER* integrates weight management programming into existing onsite preventive and clinical services as a middle school-based comprehensive wellness program. The initiative was developed to address health disparities and treat obesity among adolescents from a low-income minority community [27, 36]. The program leverages partner stakeholders’ strengths to provide programming aligned with USPSTF recommendations for moderate to high-intensity programming.

Weekly *B’N Fit POWER* initiative requirements included: 1) three 45-min (total 2.25 h) leadership sessions and 2) at least 5 h of physical activity program. The leadership sessions included the *B’N Fit POWER* leadership curriculum, cooking class, and core physical activity education conducted routinely by after-school staff without expert-level training [27]. Biweekly training sessions supported the implementation of the curriculum by after-school staff. The *B’N Fit POWER* leadership curriculum emphasized concepts supporting the attainment of “ideal” or recommended *7 Target Behaviors* based *on* American Academy of Pediatrics Expert Committee, Physical Activity Guidelines for Americans, 2nd Edition NHLBI sleep recommendations, and USDA MyPlate guidelines [11, 27, 37–39]. Attainment of target behaviors was defined as 1) eat breakfast and lunch daily; 2) eat 2–3 servings of fruits/day; 3) eat ≥3 servings of vegetables/day; 4) drink 8 cups of water and limit sugary beverages to < 1/day; 5) sleep at least 8 h/night; 6) get ≥1 h of physical activity/day, and 7) eat unhealthy snack foods and fast foods no more than weekly. The five hours of physical activity consisted of the 45-min core physical activity education leadership session plus an additional 4.25 h during the non-leadership “Physical Activity” program. Since we could not distinguish which of the 2.25 h of leadership each week consisted of the physical activity instruction, the target goal for physical activity hours per week was adjusted to 4.25 h minimum. With 36 weeks of programming a year, each student was scheduled to have 81 h a year of leadership (with 75% expected attendance at 60.75 h) and 153 h of physical activity (with 75% expected attendance at 114.75 h.)

### Student enrollment

**Fig. 1:**
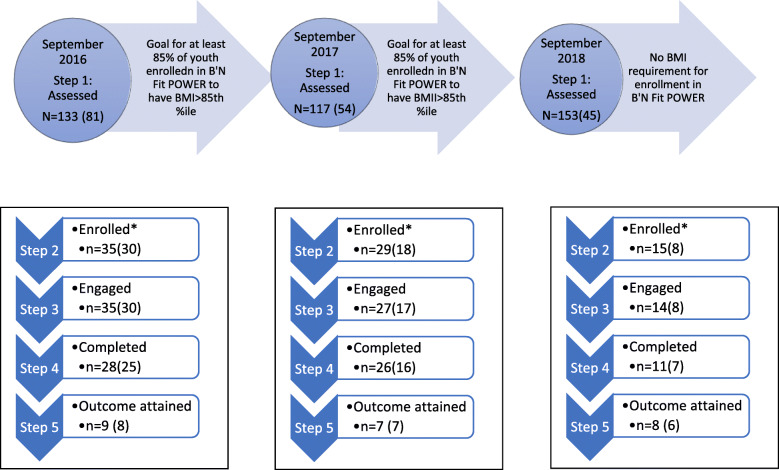
Proportions of Students Attaining Step Milestones Each School Year. *Step 1*– includes those students assessed; *Step 2* includes those students enrolled in *B’N Fit POW*ER; *Step 3*– includes those students that engaged in the program by attending at least one clinic and one after-school session; *Step 4-*includes those students that completed the program by attending all three clinics; and *Step 5 –*includes students who demonstrated either maintenance or an improvement in BMI Z-score. Number of students BMI > 85th%ile are represented within parentheses. * *B’N Fit POWER* enrollment restricted to 40 students each for 2016 and 2017 and 20 for 2018 based on staffing limitations. The numbers within the parenthesis reflect sub-group students whose BMI ≥85th percentile

In the winter before the 2016–17, 2017–18, and 2018–19 school years, the after-school program director sent an opt-out letter to the parents of all 5th–8th grade PS/MS-95 students registered in the after-school program. Parents were informed to contact the director if they did not want their child to participate in a health screening or the program. At the start of the school year, following health screening, parents were invited to enroll their children in *B’N Fit POWER* (Enhanced program) middle school-based after-school program. Inclusion criteria for a *B’N Fit POWER* invitation were students aged 11–14 years, entering the 6th to 8th grade in the fall of the upcoming school year, and interested in registering in both the after-school program and the SBHC. Students were excluded if they had a mental illness rendering them incapable of providing assent for research or complying with the after-school program protocol or had medical problems that made it unsafe for them to participate in the program [27]. The enrollment was capped at 40 students for the first two years and 20 students for the third year due to staff and resource availability constraints and programming capacity. The target enrollment initially was to recruit at least 85% of program students with a BMI >85th percentile. Enrollment in the second year dropped related to the stigma surrounding participating in a program for overweight youth. Thus, the recruitment objective was dropped for the third year. (See Fig. 1).

### Outcome metrics

The process (implementation) evaluation metrics of this study measured the proportion of students attaining the *Wellness Cascade* milestone steps, including assessment, enrollment, engagement, and *B’N Fit POWER* completion [40]. Briefly, the *Wellness Cascade* provides a construct for prevention and treatment of overweight and obesity in youth using attainment of its five-step milestones: 1) to establish the prevalence of overweight and obesity in a particular setting, 2) to foster linkages to care, 3) engagement in and 4) completion of environmentally supported obesity prevention and healthy lifestyle treatment programming, and 5) evaluation of outcomes as a tool for improving population health [39].

Outcome metrics were determined with stakeholder input considering relevant measures commonly obtained and utilized by the partners with the potential to ascertain data that would be accurate, credible, and reproducible measures of progress [27]. Primary outcomes included: 1) Height and weight collected up to four times a year either by SBHC nursing staff during routine clinical assessments or by the school’s Physical Education staff as part of the Fitnessgram® annual health screening [41]. BMI’s derived were categorized into four groups a) normal if BMI >5th and < 85th %ile; b) overweight if BMIs ≥85th and < 95th %ile; c) obese if BMIs ≥95th and < 99th %ile; and d) severely obese if BMIs ≥99th %ile [42]. 2) Levels of self-reported attainment of the *7 Target Behavior* attainment over the preceding week were assessed four times a school year (0,3,6 and 9 months) using the 25-item *B’N Fit POWER Target Behavior* survey embedded into different activities (Additional File 1**)**. After-school staff used student self-administered paper and electronic surveys during the annual after-school health screening and during after-school activities while SBHC clinical staff administered the survey using Epic electronic health record (EHR) flowsheets during routine clinical assessments. BMI clinic data were obtained from the Epic (EHR) database, and attendance at after-school sessions was monitored using the after-school NYC DYCD database.

### Statistical analysis

Baseline demographic, anthropometric information, and target behaviors were numerically summarized using descriptive statistics. Each target behavior received a score, where a score of “1” indicated behavior attainment (e.g. received score of “1” if consumed ≥3 servings of vegetables/day) while a score of “0” indicated no attainment (e.g. received score of “0” if consumed 2 or fewer servings of vegetables/day). We derived a summary *composite score* (range: 0 to 7), a sum of 7 binary target behavior attainments with higher scores indicating better overall behavior. The *composite score* and individual target behavior change from baseline to one academic year follow-up (9-month) were examined using the Wilcoxon signed-rank test or McNemar’s Chi-Square test as appropriate. As the target behaviors were measured repeatedly up to 4 visits, the *composite score*, individual target behavior achievement responses, and BMI Z-score trajectories over the visits were assessed using a generalized linear (e.g., composite scores, BMI Z-score) or logistic (e.g. individual target behavior) mixed-effects model (GLMM) with random intercepts for each subject, and the school year and elapsed time since the baseline as fixed effects. The differences in response trajectories between the students with low (< 75% after-school attendance) and high (≥75%) after-school attendance were assessed using GLMM models that included the elapsed time, after-school attendance (low and high), the interaction of elapsed time, and attendance and school year as fixed effects with random intercepts. The continuous linear models were estimated using a restricted maximum likelihood approach, while the nonlinear models were estimated using an adaptive quadrature estimation procedure. A subgroup analysis was done to assess how the program impacted those students (*n* = 55) with overweight (BMI >85th%ile) or obesity (BMI > 95th%ile). The statistical methods mentioned above were repeated for the subgroup analysis. For the ten students who continued in the program for more than one year, only data collected during the first year of enrollment was considered.

Missing data observed (e.g., target behavior and height and weight data were not available if subjects did not attend SBHC follow-up visits randomly and not all students completed all self-administered target behavior surveys or underwent height and weight assessments as part of the after-school annual health screenings) in the study were handled using a multiple imputations (MI) approach. A fully conditional specification under missing at random assumption was considered, and 30 imputed datasets were generated using IVEWARE version 0.3 software (University of Michigan). All analyses were performed using R software version 3.6 (Vienna, Austria) [43, 44].

## Results

### Wellness Cascade milestone steps

Following the wellness cascade approach, we tracked the progress and outcomes of all available students (program effectiveness) as well as a subset of students with a BMI > 85th %ile (cascade effectiveness). Between 2016 to 2018, a total of 601 letters were sent home by the after-school program staff. Of the students, 403 had demographic data and were assessed (*n* = 198 were absent on health screening days). Three hundred and twenty-four had screening BMI data, and of those, 180 had a BMI > 85th%ile (56% of those with BMI screening data) (Step 1 - see Fig. 1**)**. A total of 79 students assented and enrolled (56 with BMI > 85th%ile) (*Step 2*) in *B’N Fit POW*ER by registering in both SBHC and the after-school program. A total of 76 students attended at least one clinic and one after-school session and thus engaged (55 with BMI > 85th%ile) (*Step 3)* in the *B’N Fit POWER* program. A total of 65 students participated at all three clinic visits and completed the program (48 had BMI > 85th %ile) (*Step 4)*. Finally, 24 students (21 with BMI > 85th%ile) demonstrated either maintenance or an improvement in BMI Z-score (*Step 5).*

### Baseline student characteristics

Table 1 summarizes students’ demographic and baseline anthropometric information in each of the three academic years (*N* = 76). Students were predominantly Hispanic/Latino (62%), 46% were girls, a majority (80%) were overweight or obese, and over half (53.6%) were obese or severely obese. At baseline, the average BMI Z-score was 1.4 (0.8). Overall, there was low attainment of target behaviors at baseline, with 21% participating in at least 7 h of physical activity weekly, 25% eating no more than one unhealthy snack or fast food meal weekly, nearly 29% reported drinking 8 cups of sugar-free beverages daily, 29% eating 3–6 servings of vegetables daily, 32% of students eating breakfast and lunch daily, 53% eating 2–3 servings of fruit daily, and 75% sleeping at least 8 h a night. The overall target behavior *composite score* of all seven behaviors was 2.5 (SD = 1.1). Baseline anthropometric information of the sub-group of 55 youth who had a BMI >85th %ile is also presented in Table 1. Aside from anthropometrics, there was no statistical difference between the baseline characteristics of the two groups.

**Table 1:** B’N Fit POWER 2016–2018 Baseline Student Characteristics

	***B’N Fit POWER*** All Students (***N*** = 76)	***B’N Fit Power*** Sub-group students ***(≥85th %ile)*** (***N*** = 55)
***Demographics***		
Number recruited in 2016	35	30
Number recruited in 2017	27	17
Number recruited in 2018	14	8
Age, yrs. (SD)	12.4 (1.0)	12.5 (1.1)
Sex, Female, %	35 (46.1)	25 (45.5)
***Ethnicity***		
Black (Non-Hispanic)	19 (25)	14 (25)
Hispanic/Latino	47 (61.8)	32 (58)
White (Non-Hispanic)	3 (3.9)	3 (5)
***Anthropometrics: x̅*** ***+*** ***SD***		
BMI, kg/m2	24.7 (3.8)	26.2 (2.3)
BMI percentile, %	86.7 (20.1)	95.1 (3.6)
BMI Z-score	1.4 (0.8)	1.7 (0.3)
***Obesity category***		
BMI (5–84.9%ile) Healthy weight	14 (20.3)	0 (0)
BMI (85–94.9%ile) Overweight	18 (26.1)	18 (32.7)
BMI (95–98.9%ile) Obesity	33 (47.8)	33 (60)
BMI (> 99%ile) Severe obesity	4 (5.8)	4 (7.3)
***Baseline Attainment of Target behaviors (%)***		
Breakfast daily	34 (45.3)	25 (46.3)
Lunch daily	43 (57.3)	29 (53.7)
Breakfast and Lunch daily	24 (32)	17 (31.5)
Fruit (2–3 servings/day)	40 (53.3)	29 (53.7)
Vegetable (3–6 servings/day)	22 (29.3)	16 (29.6)
Sugarfree Beverages (8 cups a day)	21 (28.8)	15 (28.8)
Sugary Beverages (< 1 cup a day)	20 (26.7)	15 (27.8)
Sugarfree and Sugary Beverages (8 cups & < 1 cup a day)	7 (9.3)	5 (9.3)
Sleep (at least 8 h a night)	56 (74.7)	41 (75.9)
Physical activity (≥1 h of physical activity/day)	16 (21.3)	12 (22.2)
Unhealthy snacks (no more than weekly)	20 (27)	16 (29.6)
Fast foods (no more than weekly)	53 (70.7)	37 (68.5)
Unhealthy snack and fast foods (no more than weekly)	19 (25.3)	15 (27.8)
***Composite score (SD),*** **range: 0–7**	2.5 (1.1)	2.5 (1.1)

### Afterschool program and clinic attendance

The overall after-school leadership and physical activity attendance following one academic year were 63.7 (+ 23.6) hours and 67.9 (+ 39.3) hours, respectively. The students participated less than expected– 1.8 h per week in leadership (80% of expected 2.25 h) and 1.9 h per week in physical activity (45% of expected 4.25 ). Participation at clinic visits was high, with 85.5% (65/76) attending all three visits. Yet, participation in all three clinic visits and at least 75% attendance at leadership and physical activity was low at 15.8% (12/76).

### BMI Z-score and composite target behavior score changes after participation in B’N fit POWER

**Fig. 2:**
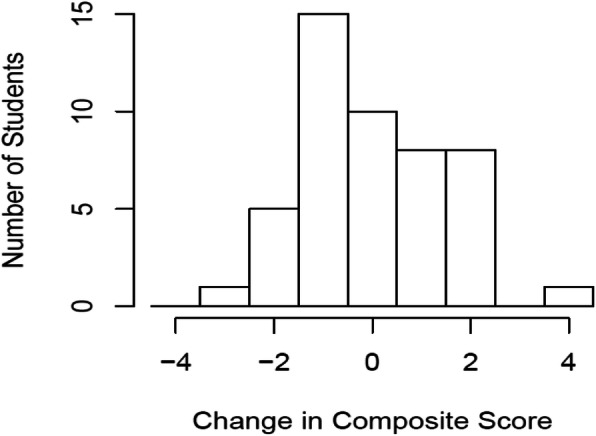
Composite Target Behavior Score Changes After a Year (*N* = 76)

Comparing baseline and 4th visits, students engaged in the *B’N Fit POWER* program showed a negligible change in BMI Z-score of 0.02 (SE = 0.03, *p* = 0.29). Multiple imputations (MI) analysis showed a similar result of − 0.04 (SE = 0.15). Similarly, we did not observe any significant change in composite score over time (mean change = 0, SE = 0.21, *p* = 0.96) (Fig. 2). The BMI Z-score change in the sub-group students was 0.01 (SE: 0.04, *p =* 0.49), while not statistically significant, the difference in composite score was − 0.22 (SE: 0.24, *p* = 0.47), suggesting a potential narrowing of uptake of expected negative behaviors by the highly-impacted group.

**Fig. 3:**
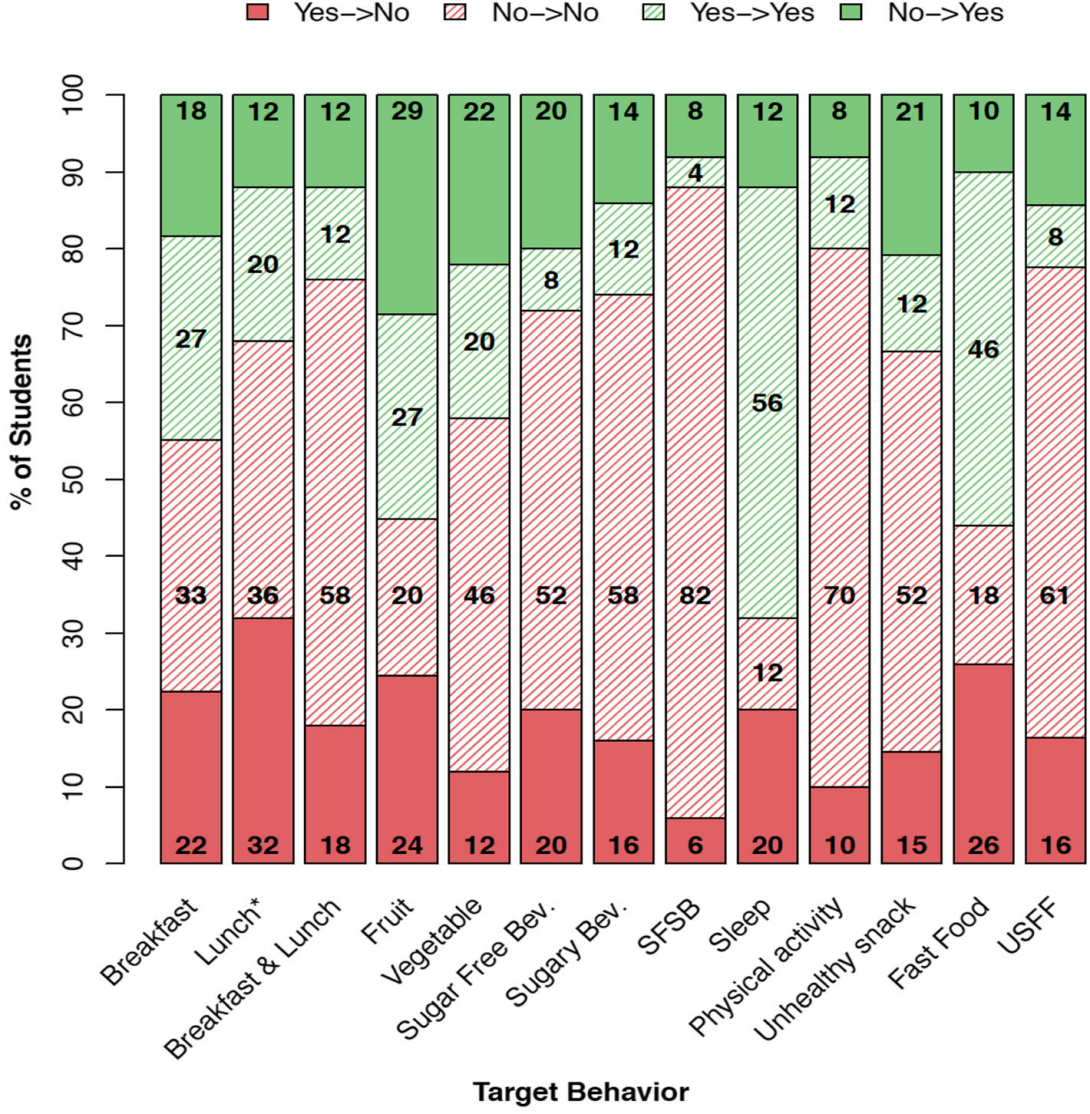
Proportions of Students Attaining Individual Target Behaviors After One Year (N = 76). Sugar-Free Bev. –Sugar-free beverage consumption; Sugary Bev. –Sugary beverage consumption; SFSB –Sugar-free beverage and sugary beverage consumption; USFF – Unhealthy snack food and fast food consumption. *p = 0.05 by McNeMar’s test. Note: Target behaviors attainment standards according to American Academy of Pediatrics Expert Committee, NHLBI sleep recommendations, Physical Activity Guidelines for Americans 2nd Edition, and USDA MyPlate guidelines

Assessment of changes in each target behavior showed progress in some target behaviors **(**Fig. 3**)**: fruit and vegetable consumption, combined increased consumption of sugar-free beverages with decreased sugary drinks, and improved unhealthy snack food consumption following participation in the program. Students, however, lacked progress in breakfast, lunch (*p =* 0.05), fast food, sugary beverage consumption, and target sleep hours. Similarly, multiple imputation results suggested a marginally significant increase in vegetable consumption (*p* = 0.07), a decrease in breakfast consumption (*p* = 0.09), and an increase in eating fast food (p = 0.05). Individual behavioral changes in the sub-group students suggested similar positive progress in percentages as in the total program students. (Additional File 2***-*** Supplemental Figure1).

The target behavior trajectory changes showed a positive trend, though not statistically significant. Specifically, the composite score suggested progress in vegetable and fruit consumption, sugar-free and sugary beverage consumption, physical activity, and unhealthy snack consumption. (Additional File 2 -Supplemental Table 1). Multiple imputation results showed similar magnitudes of improvement in the target behaviors mentioned above except for the composite score, fruit consumption, and physical activity.

The students with > 75% after-school attendance at leadership (which included nutrition education and cooking classes) and physical activity had a higher tendency (though not statistically significant) for target behavior improvement than those with lower attendance. The improvements included a 3.6 fold increase in fruit consumption and a 4.2 fold increase in drinking less sugary beverages, compared to students with lower after-school attendance (< 75%) **(**Table 2***).*** Multiple imputation results showed similar results. The sub-group students who had higher after school attendance showed progress in BMI Z-score, fruit (OR: 5.93, 95%CI: 0.85, 41.46), and vegetable (OR: 1.62, 95% CI: 0.11, 23.02) consumption. They also showed improved progress in physical activity and eating less unhealthy snacks target behavior attainment. (Additional File 2 -Supplemental Table 2).

**Table 2:** Target behavior change for Low (< 75%) and High (> 75%) After-school Attendance ^a, b^

	Afterschool Attendance					
	Low ***N*** = 56 (78%)			High ***N*** = 16 (22%)		
	Mean attendance (3.1 h/week)			Mean attendance (5.7 h/week)		
**Outcome**	**β per one academic year**	**95% CI**	***P*-value**	**β per one academic year**	**95% CI**	***P*-value**
Composite Score^a^	0.07	(−0.37–0.51)	0.745	0.16	(− 0.65–0.97)	0.700
BMI Z-score	0.00	(− 0.05–0.06)	0.923	0.02	(− 0.08–0.12)	0.632
**Behavior**	**OR per one academic year**	**95% CI**	***P*-value**	**OR per one academic year**	**95% CI**	***P*-value**
Breakfast	0.80	(0.23–2.77)	0.728	0.96	(0.12–7.69)	0.969
Lunch	0.37	(0.11–1.24)	0.108	0.00	(0–0.1)	0.001
Breakfast & Lunch	0.88	(0.26–2.9)	0.828	0.30	(0.03–3.48)	0.334
Fruit	0.98	(0.42–2.28)	0.965	3.55	(0.65–19.29)	0.143
Vegetable	2.07	(0.67–6.37)	0.204	2.22	(0.24–20.79)	0.484
Sugar Free Beverage	1.09	(0.35–3.32)	0.886	0.90	(0.12–6.73)	0.922
Sugary Beverage	0.63	(0.2–1.91)	0.411	2.64	(0.31–22.59)	0.376
Sugar-Free and Sugary Beverage	1.13	(0.25–5.09)	0.877	1.83	(0.12–28.91)	0.669
Sleep	0.83	(0.25–2.75)	0.762	0.24	(0.03–1.92)	0.179
Physical activity	1.09	(0.28–4.22)	0.905	1.59	(0.15–16.35)	0.698
Unhealthy snacks	1.25	(0.46–3.4)	0.660	1.55	(0.21–11.6)	0.668
Fast Food	0.55	(0.19–1.58)	0.268	0.38	(0.07–2.19)	0.281
Unhealthy Snack and Fast Food	0.77	(0.27–2.18)	0.627	0.82	(0.07–9.33)	0.870

^a^- 4 students were missing after-school attendance data

^b^ ***-*** A generalized linear (for the outcomes such as composite score and BMI Z-score) or logistic (individual target behavior) mixed-effects model was fitted including the school year, elapsed time, afterschool attendance (low and high) and an interaction of elapsed time and attendance as fixed effects and random intercepts by subject. Then, the linear trend by elapsed time (per one academic year as 9 months) according to afterschool attendance (low, high) was calculated based on the model fit

### Associations between target behavior change and BMI Z-score

Although not significant, improvements in individual target behaviors and the *composite score* showed progress in BMI Z-score reduction. This was seen mainly in those students who had accomplished the target sleep hours with a statistically significant decrease in BMI Z-score (β = − 0.05, 95% CI -0.1 – − 0.003, *p* = 0.038) (Table 3***).*** The subgroup analyses showed similar trends similar to the whole program participants, especially concerning eating breakfast, which showed a significant reduction in BMI Z score (β: -0.06, 95% CI: − 0.11, − 0.01, *p* = 0.02*). (*Additional File 2*-*Supplemental Table 3).

**Table 3:** Association Between Target Behavior Attainment as an Exposure and BMI Z-score^a^

Exposure	Β	95% CI	***P***-value
Composite score*	−0.01	(−0.03, 0.01)	0.367
Breakfast	−0.04	(−0.09, 0.02)	0.196
Lunch	−0.02	(− 0.07, 0.03)	0.416
Breakfast & lunch	−0.01	(−0.07, 0.04)	0.635
Fruit	0	(−0.04, 0.04)	0.99
Vegetable	−0.03	(−0.08, 0.02)	0.248
Sugar free beverage	−0.02	(−0.07, 0.03)	0.352
Sugary beverage	0.02	(−0.03, 0.07)	0.519
Sugar free and sugary beverage	0.04	(−0.03, 0.11)	0.284
Sleep	−0.05	(−0.1, − 0.003)	**0.038**
Physical activity	−0.03	(− 0.08, 0.03)	0.374
Unhealthy snacks	0.04	(−0.01, 0.08)	0.13
Fast Food	0.02	(−0.02, 0.07)	0.32
Unhealthy snack and fast food	0.04	(−0.01, 0.09)	0.132

^a^**-** A linear mixed-effects model was fitted on the data with BMI Z-score as outcome, and school year, elapsed time, and target behavior (exposure. Each exposure one at a time in each model) as fixed effects and subject-to-subject variation by random intercepts

## Discussion

Consistent with the upward trend of more severe forms of pediatric obesity [3], and over 50% of American children today projected to be obese by the age of 35 [7], it is not surprising that 56% of this cohort demonstrated an increase in BMI z-score over one school year. Conversely, 44% of youth with overweight or obesity participating in *B’N Fit* POWER showed either maintenance or improvement in BMI z-score. We demonstrated that adding structured nutrition education and requiring a minimum number of physical activity hours for existing middle school after-school programming increases several healthy target behaviors, including students with overweight and obesity. Improvement in target behaviors, in turn, is associated with positive changes in BMI z-score. We document that participation in any structured healthy lifestyle programming may positively impact improvements in behaviors and BMI z-score. Further, students with higher after-school attendance (> 75%) had higher composite score improvement, greater BMI Z scores, and target behavior improvements (in most behaviors) compared to students with lower after-school attendance.

Given the secular trends among American youths concerning unhealthy snacking, increasing fast food consumption, declining sleep hours, and rising levels of sugary beverage consumption among black and Hispanic youth [45–49], there is an expressed need by low-income ethnic minorities community for healthy lifestyles programming to counteract these trends. Our institution’s recent community needs assessment indicated that top community-member priorities include food and nutrition concerns and obesity. The community-recommended response actions include the need for activities that increase access to healthier food, exercise, and weight loss programs [32]. Like other after-school programs that promote nutritional eating guidelines, we have demonstrated that attendance at structured nutrition education and physical activity hours is associated with reducing BMI Z-scores [21–24]. Unlike other studies that focus on after-school programming among the more general population, in low-income youth, or in ethnic minority populations, we have demonstrated a positive behavior change trend in a predominantly overweight and obese cohort of impoverished minority youth most at risk for future health disparities. A majority of the reviewed studies focused on youth in less disadvantaged communities, and although youth with minorities were studied, they had lower rates of overweight and obesity [21–24].

Like the upward secular trend of unhealthy behaviors, our adolescent cohort lacked progress in improving breakfast and lunch, fast food, sugary beverage consumption, and target sleep hours over the year that they attended the program. Our cohort’s findings may be related to adolescent rites of passage associated with increasing autonomy, independence, and peer influence among middle school students. Increasing water or sugar-free beverage consumption may be easier to attain than decreasing sugary beverage consumption given the high availability of sugary beverages relative to bottled water beverages and the relative ease of adopting a new healthy behavior compared to stopping an unhealthy behavior [50]. Further, the longstanding poverty stigma of school lunch, and students not wanting to be seen as poor, may explain a tendency for students to forego lunch and opt for fast food meals after-school [51]. The consumption of sugary beverages and fast food likely occurs once students leave school and as students become older with increasing independence to purchase food and beverages outside the school or home.

### Limitations and lessons learned

Study limitations relate predominantly to two areas: 1) availability of onsite staff to implement program components discretely and their impact on the study design, and 2) challenges in using existing data sources to evaluate program impact.

The implementation of the enhanced *B’N Fit POWER* program after-school activities was conducted routinely by after-school staff without expert-level training. There were inadequate resources to ensure standardized implementation of the curriculum by youth leaders. Also, while the abstraction of existing EHR clinical BMI data and target behavior was routine, the abstraction of target behavior data and attendance collected during after-school hours was more challenging. After-school program activity attendance data did not distinguish which leadership attendance hours each week consisted of physical activity attendance, and adjustments in daily activities reflecting individual student preferences were not recorded. For example, a student scheduled for basketball may decide to attend art that day with their friend after being marked present for the basketball session. This flexibility may be a reason that overall physical activity attendance was only 38% of expected. Conversely, all after-school program students were required to remain with their assigned leadership group for leadership sessions throughout the year, making it easier to track attendance at leadership sessions. The imposition of a more consistent and less flexible schedule for the *B’N Fit POWER* students with a higher physical activity attendance requirement may contribute to significantly more hours of physical activity attendance moving forward.

An important lesson learned relates to basing target behavior goals on “ideal” or recommended target behaviors. Given that our baseline behaviors in this cohort were far from ideal, with a composite score of 2.5 out of 7 at baseline, the target behaviors’ attainment may have been unrealistic to expect at this point. In this instance, any improvement that was less than “ideal”, even if positive, would not be assessed as a positive change. For example, with the attainment of the vegetable consumption goal set at three or more servings a day, an individual with an increase from zero servings at baseline to two servings following participation would not be assessed as attaining the target behavior. However, there is an improvement noted. The addition of personalized feedback and target behavior planning via web-based surveys and personalized reports may better capture less individual progress that supports the development of realistic target behavior goals as a first step to reaching the “ideal” target behavior goals [52].

While our ability to standardize curriculum implementation and track weekly physical activity attendance and repeated target behavior reports proved to be a challenge, we were able to demonstrate the feasibility of adding nutrition education and physical activity programming and monitoring the impact of these program elements on target behaviors changes. Future efforts to overcome these challenges include the development of user-friendly data collection infrastructures, staff trainings, and program implementation tools that promote sustained program implementation and routine monitoring.

## Conclusion

The addition of structured nutrition education and minimum required number of physical activity hours for existing middle school after-school programming with student participation in any structured healthy lifestyle programming may positively impact improvements in behaviors and BMI Z-score. These findings support the implementation of concrete guidelines to increase weekly hours of healthy lifestyle education and physical activity hours during middle school after-school programming where the majority of students may engage in recommended activities.

In an era where there continues to be a paucity of evidence for health-promoting programs in after-school settings serving ethnic minority low SES youth with overweight or obesity, our findings of overall improvements, despite the limitations, support continued program implementation [53]. Initiatives, such as the *B’N Fit POWER* intervention, targeting emerging adolescents in the middle school environment may potentially mitigate the current trajectory of adoption of unhealthy lifestyle behaviors. Such initiatives, at a point where intervention might have a positive spillover effect to promote normalization of healthy lifestyle behaviors, may ultimately flatten the obesity epidemic ravaging low-income predominantly minority communities.

## Supplementary Information

**Additional file 1: ***B’N Fit POWER Target Behavior* Survey.

**Additional file 2: **Supplementary Figures and Tables. **Supplemental Figure 1.** Proportions of Students with BMI > 85th %ile Attaining Individual Target Behaviors After One Year (*N* = 55). Sugar-Free Bev. –Sugar-free beverage consumption; Sugary Bev. –Sugary beverage consumption; SFSB –Sugar-free beverage and sugary beverage consumption; USFF – Unhealthy snack food and fast food consumption. Note: Target behaviors attainment standards according to American Academy of Pediatrics Expert Committee, NHLBI sleep recommendations, Physical Activity Guidelines for Americans 2nd Edition, and USDA MyPlate guidelines. **Supplementary Table 1.** Target Behavior Changes Following Program Participation Including Multiple Imputation (MI) Results (*N *= 76). A generalized linear (for the outcomes such as composite score and BMI Z-score) or logistic (individual target behavior) mixed-effects model was fitted including the school year, elapsed time as fixed effects and random intercepts by subject. Then, the effect of elapsed time (per one academic year as 9 months) is shown in the table. *Composite score is the score of 7 target variables. **Supplementary Table 2.** Target behavior change for Low (<75%) and High (>75%) Afterschool Attendance with BMI>85th%ile (*N *= 55). A generalized linear (for the outcomes such as composite score and BMI Z-score) or logistic (individual target behavior) mixed-effects model was fitted including the school year, elapsed time, after-school attendance (low and high) and an interaction of elapsed time and attendance as fixed effects and random intercepts by subject. Then, the linear trend by elapsed time (per one academic year as 9 months) according to after-school attendance (low, high) was calculated based on the model fit. **Supplementary Table 3.** Association Between Target Behavior Attainment as Exposure and BMI Z-score with BMI>85th%ile (*N *= 55). A linear mixed-effects model was fitted on the data with BMI Z-score as outcome, and school year, elapsed time, and target behavior (exposure. each exposure separately in a model) as fixed effects and subject-to-subject variation by random intercepts. 

## Data Availability

The datasets used and/or analyzed during the current study are available from the corresponding author on reasonable request.

## Abbreviations

SES: Socioeconomic status
USPSTF: United States Preventative Services Task Force
DM: Diabetes Mellitus
BMI: Body Mass Index
CPSTF: Community Preventive Services Task Force
B’N Fit: Bronx Nutrition and Fitness Initiative for Teens
HIPAA: *Health Insurance Portability and Accountability Act* of 1996
SBHC: School-Based Health Center
NY: New York
NYC: New York City
PS/MS: Public School/ Middle School
USDA: United States Department of Agriculture
NHLBI: National Heart, Lung, and Blood Institute
EHR: Electronic Health Record
SFSB: Sugar-free beverage and sugary beverage consumption
USFF: Unhealthy snack food and fast food consumption
