# Supplementary material for: Trends in health behavior and weight outcomes following enhanced afterschool programming participation
Source: BMC Public Health. 2021 Apr 7;21:672. doi: 10.1186/s12889-021-10700-4 (PMC8028223; doi:10.1186/s12889-021-10700-4)
Supplement: Supplementary file 1 — Additional file 1: B’N Fit POWER Target Behavior Survey. [file 12889_2021_10700_MOESM1_ESM.docx]

***Date:__________________________***

***Student ID#:________________________***

Thank you for agreeing to complete the *B’N Fit POWER* survey!

The questions you are about to complete will help us to better understand your knowledge about healthy food and physical activity and behaviors that can affect your health. They will also help us to understand your attitudes about your health and confidence in your ability to be healthy. Please answer every question carefully. If something is unclear or you have a question, please ask one of the staff to help you. Your help with this survey is greatly appreciated. This is not a test so please be as honest as you can with your responses. There are no wrong answers.

You don’t have to participate in this survey if you don’t want to. If any of the statements make you feel uncomfortable, you do not need to respond to that statement.

Please use a blue or black pen to complete the survey. Place a check in the box for your answer. If you make a mistake, place an x through the incorrect answer and check the correct box

Thanks for your time!

*The B’N Fit POWER Team*

1. How healthy are these foods for you

|  | Very | Sometimes | Not |
| --- | --- | --- | --- |
| 1. Carrot Sticks |  |  |  |
| 1. Banana |  |  |  |
| 1. Chocolate Bar |  |  |  |
| 1. Turkey |  |  |  |
| 1. Pizza |  |  |  |
| 1. Fish |  |  |  |
| 1. Crackers |  |  |  |
| 1. Cheese |  |  |  |
| 1. Donuts |  |  |  |
| 1. Ice Cream |  |  |  |
| 1. Cheese, Lettuce & tomato sandwich |  |  |  |
| 1. Pop tarts |  |  |  |

1. How healthy or beneficial are these drinks?

|  | Very | Sometimes | Not |
| --- | --- | --- | --- |
| 1. Juice |  |  |  |
| 1. Soda |  |  |  |
| 1. Lemonade |  |  |  |
| 1. Fruit Smoothie |  |  |  |
| 1. Water |  |  |  |
| 1. Milk |  |  |  |
| 1. Chocolate Milkshake |  |  |  |

3. Eating healthy and getting regular exercise (for example, sports, dance, and playing outside) will help me feel more confident.

Strongly Agree Agree Disagree Strongly Disagree

4. If I eat healthy and get regular exercise I will feel good about myself.

Strongly Agree Agree Disagree Strongly Disagree

5. Doing everything I need to eat healthy and get regular exercise is too much to think about

Strongly Agree Agree Disagree Strongly Disagree

6. Doing everything I need to eat healthy and get regular exercise would keep me from doing things I like with my friends.

Strongly Agree Agree Disagree Strongly Disagree

7. Eating healthy and getting regular exercise would keep me at a healthy weight.

Strongly Agree Agree Disagree Strongly Disagree

8 If I were to join a program to lose weight my friends would make fun of me.

Strongly Agree Agree Disagree Strongly Disagree

9. I get embarrassed if other kids see me when I am exercising

Strongly Agree Agree Disagree Strongly Disagree

10. I am so busy I can’t fit in physical activity (for example, sports, dance, or playing outside).

Strongly Agree Agree Disagree Strongly Disagree

***ALMOST DONE!***

**Please circle the answer that applies. You may circle more than one answer if applicable.**

1. Thinking back over the last week, on average how many vegetable servings did you eat per day? 1 serving= 1 baseball-sized or fist-sized portion of cooked vegetables OR 2 fist-sized portions of raw vegetables/salad.

| 0 | 1 | 2 | 3 | 4 | 1. or more |
| --- | --- | --- | --- | --- | --- |

1. Thinking back over the last week, on average how many fruit servings did you eat per day? 1 serving= 1 baseball-sized or fist-sized portion of fresh, frozen, or canned fruit.

| 0 | 1 | 2 | 3 | 4 | 1. or more |
| --- | --- | --- | --- | --- | --- |

1. Thinking back over the last week, on average how many hours per day did you sit and watch a screen? Include time spent TV or movies, play video games, play games on your smart phone or other similar devices, use a computer or play computer games outside of school and homework.

| 0 | 1 | 2 | 3 | 4 | 1. or more |
| --- | --- | --- | --- | --- | --- |

1. How many days in the last week were you physically active for a total of 60 minutes or more? Include the total time you spent each day in any physical activity that increased your heart rate and made you breathe hard.

| 0 | 1 | 2 | 3 | 4 | 5 | 6 | 7 |
| --- | --- | --- | --- | --- | --- | --- | --- |

15. During the past week, on how many days did you eat breakfast?

| 0 days | 1 day | 2 days | 3 days | 4 days | 5 days | 6 days | 7 days |
| --- | --- | --- | --- | --- | --- | --- | --- |

15a. During the past week, on how many days did you eat breakfast provided by your school?

| 0 days | 1 day | 2 days | 3 days | 4 days | 5 days |
| --- | --- | --- | --- | --- | --- |

16. During the past week, on how many days did you eat lunch?

| 0 days | 1 day | 2 days | 3 days | 4 days | 5 days | 6 days | 7 days |
| --- | --- | --- | --- | --- | --- | --- | --- |

16a. During the past week, on how many days did you eat lunch provided by your school?

| 0 days | 1 day | 2 days | 3 days | 4 days | 5 days |
| --- | --- | --- | --- | --- | --- |

17. Thinking back over the last week, on average how many small (8 oz). cups of water or other sugar free beverages did you drink PER DAY? Examples include seltzer or sparkling water, unsweetened tea or coffee, and any “diet”, “zero”, or “light” drinks.

| 0 | 1 | 2 | 3 | 4 | 5 | 6 | 7 | 1. or more |
| --- | --- | --- | --- | --- | --- | --- | --- | --- |

18. During the past week, how many times PER DAY did you drink sugar-sweetened drinks such as soda ( including ginger-ale,) sports drinks (such as Gatorade or powerade), 100% fruit juices ( such as orange, apple, or grape juice) energy drinks, fruit punch, fruit-flavored drinks, or sugar-sweetened teas such as Arizona Ice Tea or non-diet Snapple,? (Do not count diet or sugar free drinks.)

| 0 | 1 | 2 | 3 | 4 | 5 | 6 | 7 | 1. or more |
| --- | --- | --- | --- | --- | --- | --- | --- | --- |

19. During the past week, how many times did you eat food from a fast food restaurant or food cart/truck (ex. McDonalds, Wendy's, KFC, a pizzeria, hot dog stand)?

| I did not eat food from a fast food restaurant, during the past week | 1 time during the past week | 2 to 3 times during the past week | 4 to 6 times during the past week | 1 time a day | 2 times a day | 3 times a day | 4 or more times a day |
| --- | --- | --- | --- | --- | --- | --- | --- |

20. During the past week, how many times did you eat unhealthy snacks foods, including potato chips, cookies, doughnuts, cakes or candy?

| I did not eat unhealthy snack foods during the past week | 1 time during the past week | 2 to 3 times during the past week | 4 to 6 times during the past week | 1 time a day | 2 times a day | 3 times a day | 4 or more times a day |
| --- | --- | --- | --- | --- | --- | --- | --- |

21. On an average school night, how many hours of sleep do you get?

| 4 or less hours | 5 hours | 6 hours | 7 hours | 8 hours | 9 hours | 10 hours or more |
| --- | --- | --- | --- | --- | --- | --- |

1. Of the behaviors that you are NOT currently practicing, which behavior would you be most interested in or most willing to work towards achieving TODAY? Circle one.

| Eat breakfast and lunch everyday | Eat 2-3 servings of fruits everyday | Eat 3-6 servings of vegetables everyday | Eat unhealthy snack foods or fast foods no more than once a week | Drink 8 cups or more of water or sugar-free beverages and limit sugary drinks to less than 1 small cup per day | Get 9 hours of sleep every night | Get at least 1 hour of physical activity everyday | None of these |
| --- | --- | --- | --- | --- | --- | --- | --- |

***Finally…***

**Please circle the answer that applies. You may circle more than one answer if applicable.**

23. How would you describe yourself as a student? Would you say .....

[1] Excellent

[2] Good

[3] Fair

[4] Poor

24. What was your overall average last marking period n school?

[1] A (either A-, A or A+ ; 90-100)

[2] B (either B-, B or B+; 80-89)

[3] C (either C-, C or C+; 70-79)

[4] D (either D-, D or D+; 65-69)

[5] F (64 and under)

[6] Don’t know

[7[ Other

25. During the last marking period, about how many days were you absent from school?

____ ___ (days)

***ALL DONE – THANK-YOU!***
