# Supplementary material for: Trends in health behavior and weight outcomes following enhanced afterschool programming participation
Source: BMC Public Health. 2021 Apr 7;21:672. doi: 10.1186/s12889-021-10700-4 (PMC8028223; doi:10.1186/s12889-021-10700-4)
Supplement: Supplementary file 2 — Additional file 2: Supplementary Figures and Tables. Supplemental Figure 1. Proportions of Students with BMI > 85th %ile Attaining Individual Target Behaviors After One Year (N = 55). Sugar-Free Bev. –Sugar-free beverage consumption; Sugary Bev. –Sugary beverage consumption; SFSB –Sugar-free beverage and sugary beverage consumption; USFF – Unhealthy snack food and fast food consumption. Note: Target behaviors attainment standards according to American Academy of Pediatrics Expert Committee, NHLBI sleep recommendations, Physical Activity Guidelines for Americans 2nd Edition, and USDA MyPlate guidelines. Supplementary Table 1. Target Behavior Changes Following Program Participation Including Multiple Imputation (MI) Results (N = 76). A generalized linear (for the outcomes such as composite score and BMI Z-score) or logistic (individual target behavior) mixed-effects model was fitted including the school year, elapsed time as fixed effects and random intercepts by subject. Then, the effect of elapsed time (per one academic year as 9 months) is shown in the table. *Composite score is the score of 7 target variables. Supplementary Table 2. Target behavior change for Low (<75%) and High (>75%) Afterschool Attendance with BMI>85th%ile (N = 55). A generalized linear (for the outcomes such as composite score and BMI Z-score) or logistic (individual target behavior) mixed-effects model was fitted including the school year, elapsed time, after-school attendance (low and high) and an interaction of elapsed time and attendance as fixed effects and random intercepts by subject. Then, the linear trend by elapsed time (per one academic year as 9 months) according to after-school attendance (low, high) was calculated based on the model fit. Supplementary Table 3. Association Between Target Behavior Attainment as Exposure and BMI Z-score with BMI>85th%ile (N = 55). A linear mixed-effects model was fitted on the data with BMI Z-score as outcome, and school year, elapsed time, and targ [file 12889_2021_10700_MOESM2_ESM.docx]

***Figure 1. Proportions of Students with BMI>85^th^ %ile Attaining Individual Target Behaviors After One Year (N=55)***


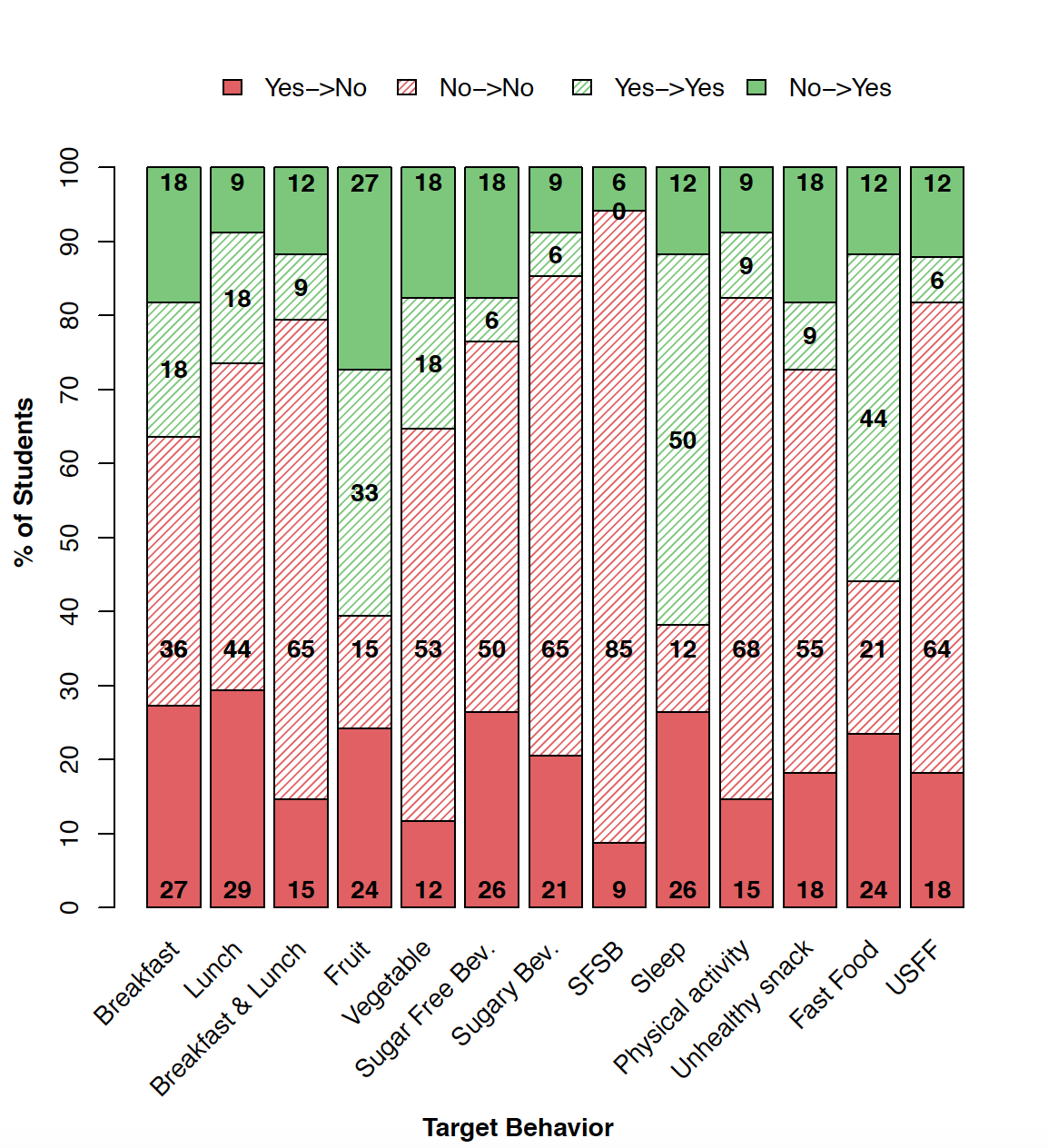


*T****able 1. Target Behavior Changes Following Program Participation Including Multiple Imputation (MI) Results (N=76)***

|  | **Beta per one academic year**  ***(MI)*** | **95% CI**  ***(MI)*** | **P-value**  ***(MI)*** |
| --- | --- | --- | --- |
| Composite Score* | 0.09  *(-0.13)* | (-0.28, 0.46*)*  *(-0.54-0.28)* | 0.632  *(0.520)* |
| BMI Z-score | 0.01  *(-0.04)* | (-0.04, 0.05*)*  *(-0.45-0.37)* | 0.799  *(0.838)* |
|  | **OR per one academic year** | **95% CI** | **P-value** |
| Breakfast | 1.06  *(0.49)* | (0.38-2.99)  *(0.2-1.22)* | 0.914  *(0.127)* |
| **Lunch** | 0.18  *(0.14)* | (0.06-0.52)  *(0.05-0.37)* | 0.001  ***(6.5E-05)*** |
| Breakfast & Lunch | 0.7  *(0.46)* | (0.25-1.97)  *(0.18-1.17)* | 0.5  *(0.102)* |
| Fruit | 1.16  *(0.65)* | (0.56-2.42)  *(0.32-1.28)* | 0.688  *(0.211)* |
| Vegetable | 1.95  *(2.25)* | (0.72-5.27)  *(0.85-5.93)* | 0.186  *(0.101)* |
| Sugar Free Beverage | 1.25  *(1.16)* | (0.48-3.21)  *(0.48-2.79)* | 0.647  *(0.741)* |
| Sugary Beverage | 0.86  *(1.37)* | (0.32-2.3)  *(0.56-3.39)* | 0.756  *(0.487)* |
| Sugar-Free and Sugary Beverage | 1.49  *(1.45)* | (0.41-5.44)  *(0.38-5.47)* | 0.544  *(0.584)* |
| Sleep | 0.74  *(0.70)* | (0.27-2.02)  *(0.26-1.84)* | 0.558  *(0.465)* |
| Physical activity | 1.2  *(0.79)* | (0.27-2.02)  *(0.28-2.22)* | 0.756  *(0.647)* |
| Unhealthy snack | 1.32  *(1.59)* | (0.55-3.18)  *(0.68-3.73)* | 0.54  *(0.284)* |
| Fast Food | 0.5  *(0.49)* | (0.21-1.22)  *(0.21-1.15)* | 0.13  *(0.101)* |
| Unhealthy Snack and Fast Food | 0.77  *(0.91)* | (0.29-2.01)  *(0.32-2.63)* | 0.593  *(0.867)* |

**Composite score* is the score of 7 target variables

A generalized linear (for the outcomes such as composite score and BMI Z-score) or logistic (individual target behavior) mixed-effects model was fitted including the school year, elapsed time as fixed effects and random intercepts by subject. Then, the effect of elapsed time (per one academic year as 9 months) is shown in the table.

***Table 2. Target behavior change for Low (<75%) and High (>75%) Afterschool Attendance with BMI>85^th^%ile (N=55)***

|  | **Low afterschool attendance** | | | **High afterschool attendance** | | | Interaction |
| --- | --- | --- | --- | --- | --- | --- | --- |
|  | **Beta per one academic year** | 95% CI | P | **Beta per one academic year** | 95% CI | P | P |
| Composite Score | -0.18 | (-0.7-0.33) | 0.478 | 0.03 | (-0.84-0.91) | 0.938 | 0.67 |
| BMI.Z-score | -0.01 | (-0.07-0.04) | 0.676 | -0.01 | (-0.11-0.08) | 0.822 | 0.989 |
|  | **OR per one academic year** | 95% CI | P | **OR per one academic year** | 95% CI | P | P |
| Breakfast | 0.67 | (0.16-2.72) | 0.571 | 0.32 | (0.03-3.31) | 0.342 | 0.602 |
| **Lunch** | 0.36 | (0.08-1.67) | 0.190 | 0.00 | (0-0.05) | 0.002 | 0.007 |
| Breakfast & Lunch | 0.86 | (0.21-3.5) | 0.833 | 0.15 | (0.01-2.43) | 0.181 | 0.27 |
| Fruit | 0.92 | (0.35-2.41) | 0.859 | 5.93 | (0.85-41.46) | 0.073 | 0.092 |
| Vegetable | 1.51 | (0.38-6.02) | 0.558 | 1.62 | (0.11-23.02) | 0.724 | 0.965 |
| Sugar Free Beverage | 1.09 | (0.31-3.81) | 0.889 | 0.63 | (0.07-5.47) | 0.677 | 0.666 |
| Sugary Beverage | 0.29 | (0.07-1.14) | 0.076 | 2.14 | (0.23-19.58) | 0.502 | 0.131 |
| Sugar-Free and Sugary Beverage | 0.59 | (0.1-3.67) | 0.573 | 1.22 | (0.06-26.94) | 0.900 | 0.693 |
| Sleep | 0.73 | (0.2-2.61) | 0.628 | 0.23 | (0.03-1.75) | 0.156 | 0.344 |
| Physical activity | 0.90 | (0.21-3.84) | 0.892 | 1.70 | (0.16-18.42) | 0.664 | 0.661 |
| Unhealthy snack | 0.63 | (0.19-2.05) | 0.443 | 1.81 | (0.24-13.61) | 0.563 | 0.374 |
| Fast Food | 0.71 | (0.21-2.42) | 0.579 | 0.49 | (0.08-3.13) | 0.450 | 0.747 |
| Unhealthy Snack and Fast Food | 0.49 | (0.15-1.65) | 0.252 | 0.85 | (0.07-9.94) | 0.896 | 0.698 |

A generalized linear (for the outcomes such as composite score and BMI Z-score) or logistic (individual target behavior) mixed-effects model was fitted including the school year, elapsed time, after-school attendance (low and high) and an interaction of elapsed time and attendance as fixed effects and random intercepts by subject. Then, the linear trend by elapsed time (per one academic year as 9 months) according to after-school attendance (low, high) was calculated based on the model fit.

***Table 3. Association Between Target Behavior Attainment as Exposure and BMI Z-score with BMI>85^th^%ile (N=55)***

|  | Beta | 95% CI | P |
| --- | --- | --- | --- |
| Composite Score | -0.009 | (-0.027, 0.009) | 0.338 |
| Breakfast | -0.057 | (-0.105, -0.008) | **0.024** |
| **Lunch** | -0.025 | (-0.074, 0.025) | 0.327 |
| Breakfast & Lunch | -0.05 | (-0.101, 0.001) | **0.056** |
| Fruit | 0.006 | (-0.031, 0.044) | 0.743 |
| Vegetable | -0.022 | (-0.073, 0.028) | 0.398 |
| Sugar Free Beverage | -0.006 | (-0.052, 0.041) | 0.81 |
| Sugary Beverage | -0.011 | (-0.062, 0.039) | 0.661 |
| Sugar-Free and Sugary Beverage | 0.004 | (-0.066, 0.075) | 0.909 |
| Sleep | -0.049 | (-0.094, -0.003) | **0.036** |
| Physical activity | -0.016 | (-0.069, 0.036) | 0.552 |
| Unhealthy snack | 0.038 | (-0.006, 0.083) | **0.097** |
| Fast Food | 0.029 | (-0.016, 0.074) | 0.215 |
| Unhealthy Snack and Fast Food | 0.061 | (0.014, 0.107) | **0.012** |

A linear mixed-effects model was fitted on the data with BMI Z-score as outcome, and school year, elapsed time, and target behavior (exposure. each exposure separately in a model) as fixed effects and subject-to-subject variation by random intercepts.
